# The Extratropical Northern Hemisphere Temperature Reconstruction during the Last Millennium Based on a Novel Method

**DOI:** 10.1371/journal.pone.0146776

**Published:** 2016-01-11

**Authors:** Pei Xing, Xin Chen, Yong Luo, Suping Nie, Zongci Zhao, Jianbin Huang, Shaowu Wang

**Affiliations:** 1 Center for Earth System Science, Tsinghua University, Beijing, China; 2 Beijing Municipal Climate Center, Beijing, China; 3 National Climate Center, China Meteorological Administration, Beijing, China; 4 Department of Atmospheric Sciences, School of Physics, Peking University, Beijing, China; 5 Joint Center for Global Change Studies (JCGCS), Beijing, China; Universidade de Vigo, SPAIN

## Abstract

Large-scale climate history of the past millennium reconstructed solely from tree-ring data is prone to underestimate the amplitude of low-frequency variability. In this paper, we aimed at solving this problem by utilizing a novel method termed “MDVM”, which was a combination of the ensemble empirical mode decomposition (EEMD) and variance matching techniques. We compiled a set of 211 tree-ring records from the extratropical Northern Hemisphere (30–90°N) in an effort to develop a new reconstruction of the annual mean temperature by the MDVM method. Among these dataset, a number of 126 records were screened out to reconstruct temperature variability longer than decadal scale for the period 850–2000 AD. The MDVM reconstruction depicted significant low-frequency variability in the past millennium with evident Medieval Warm Period (MWP) over the interval 950–1150 AD and pronounced Little Ice Age (LIA) cumulating in 1450–1850 AD. In the context of 1150-year reconstruction, the accelerating warming in 20^th^ century was likely unprecedented, and the coldest decades appeared in the 1640s, 1600s and 1580s, whereas the warmest decades occurred in the 1990s, 1940s and 1930s. Additionally, the MDVM reconstruction covaried broadly with changes in natural radiative forcing, and especially showed distinct footprints of multiple volcanic eruptions in the last millennium. Comparisons of our results with previous reconstructions and model simulations showed the efficiency of the MDVM method on capturing low-frequency variability, particularly much colder signals of the LIA relative to the reference period. Our results demonstrated that the MDVM method has advantages in studying large-scale and low-frequency climate signals using pure tree-ring data.

## Introduction

Knowledge of climate change in the past is essential for understanding the response of the climate system to natural and anthropogenic forcings, and also useful for evaluating the observed 20^th^ century warming objectively in a long-term perspective. As the worldwide instrumental records rarely extend back further than the mid-19^th^ century, pre-instrumental data of temperature is often deduced from proxy records derived from natural archives. In recent years, a number of proxy-based high-resolution temperature reconstructions on hemispheric scale have been carried out (e.g. [1–12]). Nevertheless, a great range has been observed in the reconstructed amplitudes among the currently existing temperature reconstructions for the Northern Hemisphere (NH). In particular, reconstructions based solely on tree-ring data are prone to underestimate the temperature amplitude during the Medieval Warm Period (MWP) and the Little Ice Age (LIA) (e.g. [3]). In order to better capture the low-frequency variability, lower temporal resolution proxies (e.g. ice cores, sporopollen, speleothems and lake sediments, etc.) are usually used to combine with tree-ring data for large-scale temperature reconstructions (e.g. [7, 12]). However, the quality of these non-annually resolved records is hard to assess since they are difficult to calibrate against the annual meteorological data. Thus, the combination of tree ring and other proxies in low resolution may bring about significant uncertainties in the final reconstructions [13, 14].

Fortunately, some recent studies have demonstrated the feasibility of reflecting longer-term temperature trends using pure tree-ring data, provided that data are appropriately processed in respects of detrending in chronology developing, data screening, and statistical techniques [4, 10, 15, 16]. Firstly, it is known that different detrending methods usually result in obvious differences of the final chronologies in signal extracting. Compared with traditional data-adaptive curve-fitting methods (e.g. negative exponential curve or smoothing spline), the superiorities of Regional Curve Standardization [17] and signal-free method [18] have been fully demonstrated. Both methods can avoid the “segment length curse” [19], and capture more low-frequency variance in excess of the mean length of individual samples used in chronology development. Secondly, with regard to data screening, some criteria (e.g. length of the candidate chronologies, and threshold of the correlation with local/large-scale instrumental series) are usually employed in the screening process to ensure that the temperature signal is as strong as possible in the final large-scale temperature reconstructions [4, 5, 7, 9]. Previous research definitely emphasized the importance of the quality of available proxy data rather than quantity for further robustness and improvements in large-scale proxy-based reconstructions [20]. Nevertheless, as the criteria of screening procedure are more thorough and strict, fewer records could pass the screening process, easily leading to uneven spatial distribution of the tree-ring data and relatively poor spatial representativeness of the final reconstructions [1]. Thirdly, in terms of the statistical techniques used for calibration in large-scale reconstructions, the traditional method is based on the linear regression model (ordinary least squares). There are other two main methods based on variance matching (e.g. [8, 21]) and error-in-variables (EIV) regression model (total least squares) (e.g. [9, 22, 23]). The comparisons of considerable reconstructions have suggested that the application of inverse-regression-based methods, like those used in some researches [2, 3], is apt to result in large underestimation of low-frequency NH temperature variability [15, 24]. As indicated above, it is thus important and feasible to explore an effective method for large-scale temperature reconstruction on the basis of improvements in terms of chronology developing, data screening and calibrating procedures.

In this paper, a novel method applicable to pure tree-ring data was developed in order to fully make use of the tree-ring records and better capture low-frequency variability. The method we proposed was termed “MDVM”, as its core was based on ensemble empirical mode decomposition (EEMD) and variance matching techniques. Its detailed steps would be expatiated in the following sections. The objectives of this study were (1) to introduce the MDVM method, (2) to evaluate its performance by establishing a reconstruction of the extratropical NH mean temperature for the past millennium, and (3) to analyze the characteristics of reconstructed temperatures in terms of trend, amplitude and response to external forcings.

## Data

### Instrumental data

We made use of the monthly land surface air temperature dataset (CRUTEM4) [25] for the period 1850–2000, with a horizontal resolution of 5°×5° longitude-latitude grid. The extratropical (30–90°N) land-only mean annual (January-December) temperature anomaly series relative to 1961–1990 was extracted as the target series for subsequent reconstruction.

### Tree-ring data

Considering that different detrending methods would bring about discrepancy of final chronologies especially on the low-frequency variation, tree-ring raw measurements data rather than the ready-made chronologies was compiled from the International Tree-Ring Data Bank (ITRDB, http://www.ncdc.noaa.gov/data-access/paleoclimatology-data/datasets/tree-ring). We also collected several chronologies (a few were reconstructed series) from some published literatures related on regional temperature signal. The length of these tree-ring records (width data with limited density data) was mostly longer than 800 years. Additionally, we usually utilized the most up-to-date data rather than the old records.

### Model simulation data

For further analyzing, we extracted extratropical land-only mean annual simulated surface temperature from the outputs of 7 climate models for Last Millennium experiment in CMIP5 (Phase 5 of the Coupled Model Intercomparison Project) [26]: CCSM4 [27], HadCM3 [28], MPI-ESM-P [29], FGOALS-s2 [30], BCC-CSM1.1 [31], IPSL-CM5A-LR [32] and CSIRO-MK3L-1.2 [33]. These results are available via the Earth System Grid Federation (http://pcmdi9.llnl.gov/esgf-web-fe/). As described in CMIP5, the Last Millennium transient simulation is conducted by coupled climate system model (or earth system model) with external forcings and uniformly spans from 850 to 1850 AD. The simulated series were simply adjusted by removing their respective mean of the whole period (850–1850 AD) for subsequent comparison with the final reconstructed NH temperature.

## Methods

### Chronology development

The signal-free Regional Curve Standardization (SF-RCS) method [18] was used for the collected tree-ring raw measurements data. In each sampling site, the individual series were aligned by cambial age to estimate the site-specific mean biological growth curve. It has been borne out that SF-RCS is adept at preserving the low-frequency trends and can better mitigate the trend distortion problem by several recent studies on regional climate reconstructions [34–36]. The reliable period of each chronology we established was determined by expressed population signal (EPS), and the chronologies were truncated when their EPS values were less than 0.80 in consecutive 40 years [37].

### MDVM method

**Fig 1:**
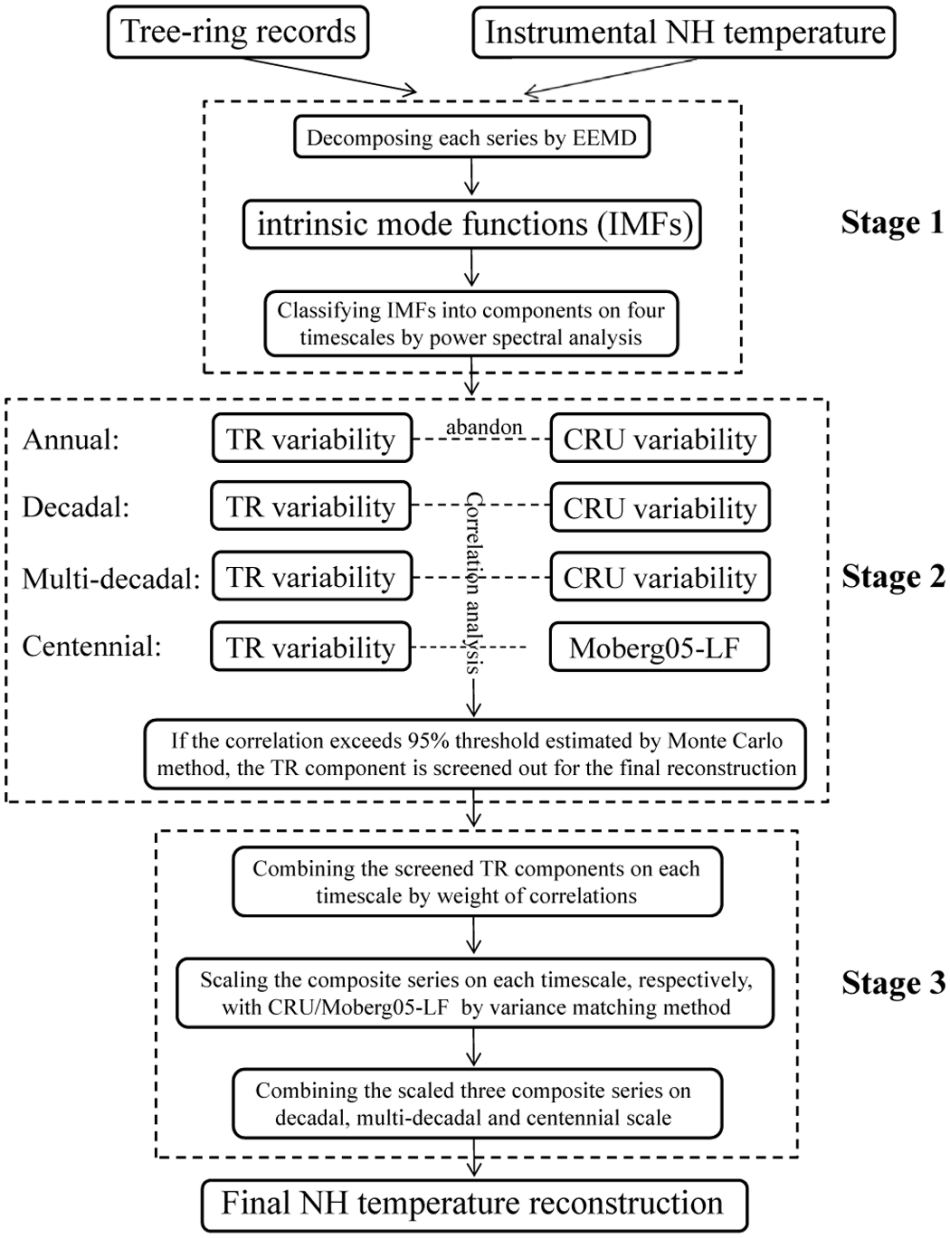
Flow chart illustrating the MDVM method developed in this study for the extratropical Northern Hemisphere temperature reconstruction.

The two keys of MDVM method are ensemble empirical mode decomposition (EEMD) and variance matching. EEMD is a data self-adapted signal processing approach, which can relief the mode-mixing problem and bring about more stable and physically interpretable results [38]. EEMD is a powerful tool for decomposing complicated dataset into a finite number of monocomponent intrinsic mode functions (IMFs). Through large numbers of trials to add white noise, only the persistent surviving IMFs could be extracted, which should contain more physical meanings [38]. EEMD has been widely used in climate research [39–41] and also applied in a few dendrochronological studies [42, 43]. Variance matching method was employed in the calibration to adjust the mean value and variance agreeing with the target series over the common period [8, 21]. It has been proved that variance matching is able to avoid the problem with underestimation of low-frequency variability associated with direct-regression-based calibration methods [15, 24]. Some researches pointed out that the indirect-regression method can avoid this problem in more effective way, and this variance matching method should fall between the direct- and indirect- regression methods [6, 24]. As shown in Fig 1, the itemized steps of MDVM method were as follows:

#### Stage 1. Decompose each series into four components on annual, decadal, multi-decadal and centennial timescales, respectively

The instrumental extratropical NH mean temperature and each tree-ring chronology (or reconstructed series) was decomposed into IMFs using the EEMD method, respectively [Eq (1)]. Then, the power spectral analysis was applied for each IMF and the residual series to diagnose the dominant period, upon which to classify the IMFs (or residual series) into certain components on four typical timescales, i.e. annual scale component (less than 10 year), decadal scale component (10–30 year), multi-decadal scale (30–90 year) and centennial scale component (longer than 90 year) [Eq (2)].

$$Ser_{i}(t)=\sum_{h=1}^{N_{i}}imf_{h}(t)+residual$$ (1)

$$Ser_{i}(t)=\sum_{j=1}^{4}comp_{i}{}^{j}(t)$$ (2)

$$Ser_{i}{}^{\prime }(t)=\sum_{j=2}^{4}comp_{i}{}^{j}(t)$$ (3)

where *N*_*i*_ was the total number of IMFs for *i*-th proxy (or instrumental CRU) time series [*Ser*_*i*_(*t*)]. Subscript *j* denoted the timescale of each component [*comp*_*i*_(*t*)]: *j* = 1 for annual scale, *j* = 2 for decadal scale, *j* = 3 for multi-decadal scale, and *j* = 4 for centennial scale.

#### Stage 2. Screen individual components with corresponding benchmark series on decadal, multi-decadal and centennial scales, respectively

Considering that the regional tree-ring record coincided with the hemispheric mean temperature mainly on decadal and longer timescales, the decomposed variability of tree ring and temperature series on annual scale was ignored in this study, which meant that the smoothed series $Ser_{i}{}^{\prime }(t)$ would consist of 3 components [Eq (3)]. Correlation analysis was employed between tree-ring and the benchmark series on decadal, multi-decadal and centennial scale, respectively. It is worth noting how to accurately estimate the threshold of significant correlation. Because the filtered time series have a reduction on the degree of freedom (DOF) comparing with the unfiltered data, more vigorous statistical tests should be utilized to demonstrate the statistical significance. In this study, we applied the Monte Carlo simulation method [44], which compared the actual correlations with correlations between instrumental temperatures and the simulated random time series generated from the original tree-ring series. The white noise was superimposed to the original series so as to generate the samples and these series would also be decomposed into components on the four timescales (details of Monte Carlo simulation please see the Supporting Information). The threshold for the value of the significant correlation coefficient was estimated using 5000 member Monte Carlo simulations with bootstrap resampling (400 trials) at a 95% level of confidence. The tree-ring component would be selected to establish the final reconstruction, if its correlation with the benchmark series on the corresponding timescale exceeded the threshold value. It was noteworthy that, owing to its relatively short length, the CRU temperature series was not able to exhibit complete signal on the centennial scale. To better reconstruct the long-term variability, an extra low-frequency series of NH temperature reconstructed in paper [8] (hereafter Moberg05-LF for short) spanning from 133 AD to 1925 AD, was used instead of CRU variability on the centennial scale, which was largely different from the traditional approach. Moberg05-LF rather than other low-frequency series was selected owing to the following two reasons. Firstly, Moberg05-LF, which was inferred from low resolution proxy records (pollen, borehole, stalagmite, etc.), was totally independent from tree-ring data. Secondly, the timescale of Moberg05-LF variability was longer than 80 year, which was similar to the centennial scale defined in this study.

#### Stage 3. Composite the screened out components, and after scaling on corresponding timescales, combine them into the final reconstruction

Weight each screened tree-ring component on the same timescale in proportion to their explained variance for the benchmark series (i.e. CRU and Moberg05-LF) [43]. Then, composite these screened out components by weight on decadal, multi-decadal and centennial scale, respectively. The function was as follows [Eq (4)]:

$$CS^{j}(t)=\frac{\overset{i=1}{\underset{N_{j}}{\sum }}\alpha \cdot r_{i,j}^{2}\cdot comp_{i}^{j}(t)}{\overset{i=1}{\underset{n}{\sum }}r_{i,j}{}^{2}}\{\begin{array}{ll} \alpha =1, & r_{i,j}>0\\ \alpha =-1, & r_{i,j}<0 \end{array}$$ (4)

where the subscript *i*, *j* denoted *i*-th standardized time series and *j*-th timescale; *N*_*j*_ was the total number of screened components at *j*-th timescale, and *CS*^*j*^(*t*) was the corresponding composite series. The weight was given by the square of each correlation coefficient (*r*_*i*,*j*_) relative to the benchmark series at *j*-th timescale.

After that three composite series were obtained, and then scale them with the benchmark series (CRU and Moberg05-LF) on the corresponding timescales, respectively, by variance matching method [Eq (5)]. Finally, combine the scaled three composite series on decadal, multi-decadal and centennial scale, and the final reconstruction of the extratropical NH mean temperature was established [Eq (6)].

$$recon^{j}(t)=\frac{(CS^{j}(t)-M_{Pcal})std_{Ical}^{j}}{std_{Pcal}^{j}}+M_{Ical}$$ (5)

$$recon_{MDVM}(t)=\sum_{j=2}^{4}recon^{j}(t)$$ (6)

where *recon*^*j*^(*t*) represented reconstructions at different timescales. *M*_*Pcal*_ and *M*_*Ical*_ were mean values of proxy composite series and instrumental series during the calibration period at *j*-th timescale. $std_{Pcal}^{j}$ and $std_{Ical}^{j}$ were the standard deviation of proxy composite series and instrumental series at *j*-th timescale.

### Validation of MDVM reconstruction

#### Pseudo-proxy experiment (PPE)

Pseudo-proxy experiment (PPE), as a more objective approach, has been widely applied to test reconstruction methods based on the climate model simulated results. The main purpose of PPE is to put the reconstruction methods to a common framework, and the model simulated temperatures can provide a longer timescale background for testing the low-frequency signal. In this research, PPE has been performed basing on the ensemble results of the CMIP5 last millennium simulations and their corresponding extended simulations. In total, seven sets of results from five models were included: BCC-CSM1.1, CSIRO-Mk3L-1.2, HadCM3, MPI-ESM-P, and GISS-E2-R (p121, p124 and p125, http://data.giss.nasa.gov/modelE/ar5/). The corresponding annual mean surface temperature of the ensemble was extracted to be the known model target.

The experiment was designed as follows. Firstly, using the bilinear interpolation scheme, the model simulation results were interpolated onto the 126 tree-ring sites (i.e. as same as the tree-ring records used for the real-world MDVM reconstruction, see S3 Table). Then Gaussian white noise with signal-to-noise ratio (SNR) of 0.1, 0.25, 0.5 and 1.0 was respectively added to the interpolated series to mimic the pseudo-proxy series over the entire 1150 years (851–2000 AD). Following the same steps of MDVM method (Stage 1–3), the variability on annual scale was discarded, and the reconstructed composite series at other three typical timescales (decadal, multi-decadal and centennial scale) were calculated basing on the pseudo-proxy series from the same proxy networks. The only difference when performing PPE was that the target series for calibration/validation was the ideal series of pure simulated NH mean surface temperature (851–2000 AD) instead. Two trials of verification tests with exchanged calibration and validation periods were conducted: Exp-A with 851–1500 AD as calibration period and 1501–2000 AD as validation period; Exp-B with 1501–2000 AD as calibration period and 851–1500 AD as validation period. Furthermore, in order to evaluate the performance of MDVM on centennial timescale, two extra metrics were calculated as well, i.e. the ratio of standard deviation of reconstructed centennial variability to simulated counterpart (low frequency stand deviation ratio, hereafter LF-Std-Ratio for short) [Eq (7)].

$$LF-Std-Ratio=std_{recon}/std_{sim}$$ (7)

#### Leave-one-out cross-validation

The “leave-one-out” cross-validation [45] was performed to verify the accuracy of MDVM reconstruction against the instrumental temperature data (CRU) over the period 1850–2000. The following statistics were examined in the validation: reduction of error (RE), coefficient of efficiency (CE), and root-mean-square error (RMSE). The uncertainty of the reconstruction was estimated using the standard deviation (*std*_*ver*_) of the instrumental extratropical NH temperature and the correlation coefficient (*r*) between the reconstructed and instrumental series in validation period [46] [Eq (8)]:

$$uncertainty=std_{ver}\cdot \sqrt{1-r^{2}}$$ (8)

Monte Carlo simulation process was also applied to test the significance of RE, CE scores and the uncertainty, and samples for the testing were generated by first-order autoregressive model (AR1) plus red noise [9].

## Results

### PPE validation and uncertainty estimation

In this study, we applied EEMD to decompose tree-ring data and instrumental series on four timescales. The tree-ring data was screened and scaled with the benchmark series on decadal, multi-decadal and centennial scale, respectively. Further details in the process of performing MDVM method including the statistical results of threshold values derived from Monte Carlo simulation, as well as the scaling and calibrating were provided in S1 and S2 Tables, S1 Fig.

**Fig 2:**
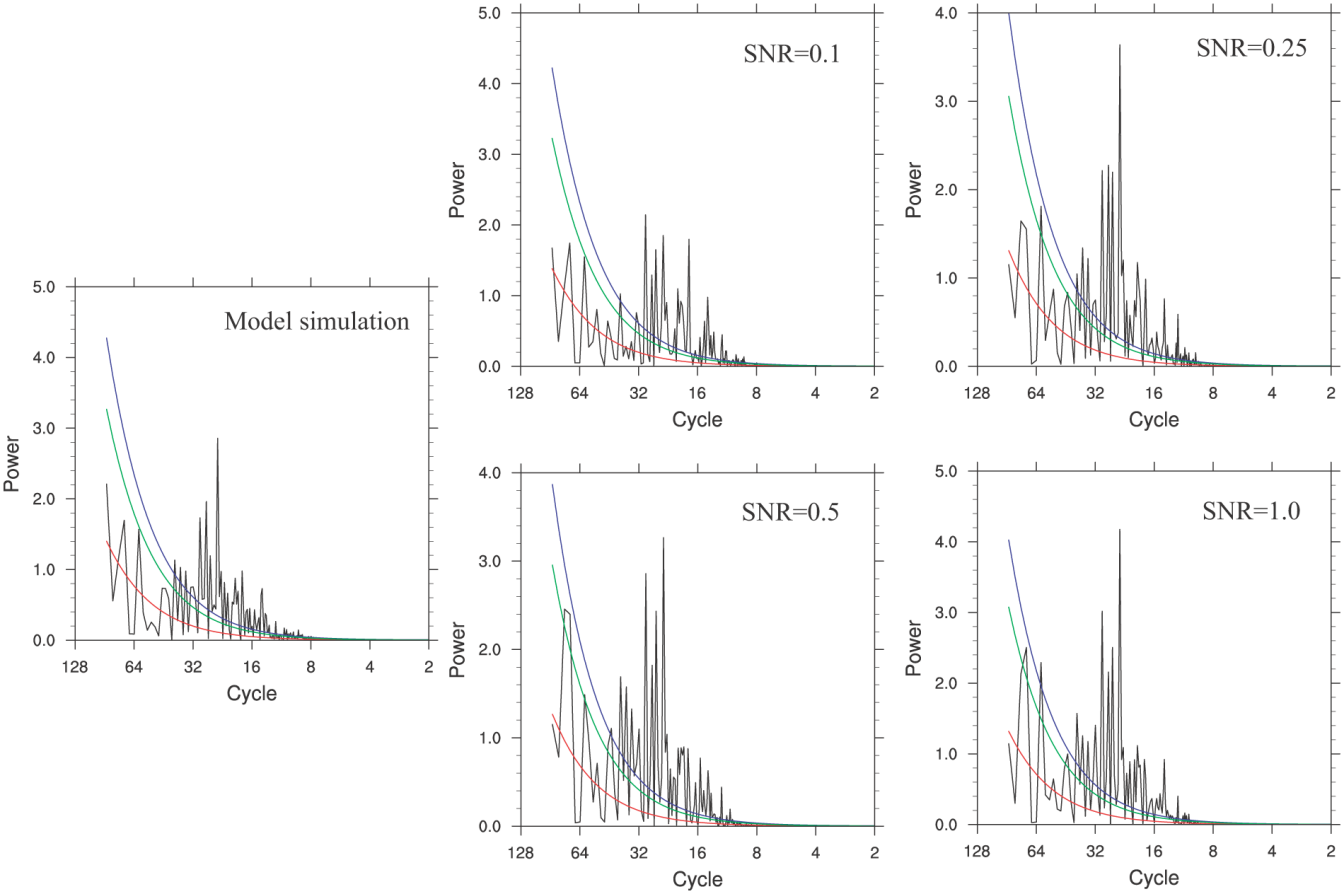
Comparison of power spectral analysis for the original model ensemble mean series and PPE reconstructed series with different SNR levels (0.1, 0.25, 0.5 and 1.0) over the common period 851–2000 AD. The red line indicated the red noise spectrum, and the blue (green) line indicated the 95% (90%) confidence level.

Statistics of split validation results for PPE was shown in Table 1. Based on the high values of RE and CE calculated from PPE test, we could basically infer a good ability of MDVM method to preserve the low-frequency signals. As levels of SNR increased, the quality of reconstruction improved gradually (i.e. the values of RE and CE increased), and the uncertainty of variability on centennial scale (Uncer-LF) basically decreased with values ranging from 0.0505 to 0.0711°C. Additionally, the LF-Std-Ratio showed different performance for Exp-A and Exp-B. In Exp-A, the values of LF-Std-Ratio were all less than 1.0 whereas the values in Exp-B were all larger than 1.0. It implied that the reconstructed centennial variability depended on the choice of calibration/validation period in some ways, owing to the different dominant external forcings driving the climate and great difference of the amplitude in the two periods (i.e. 851–1500 AD and 1501–2000 AD). For example, in Exp-B, the calibration period (1501–2000 AD) included the typical period of Little Ice Age and recent rapid warming with much larger amplitude than earlier years before 1500 AD, thus the reconstructed centennial variability in Exp-B was exaggerated to some extent compared with the known ensemble models target. In addition, the verification series of Exp-A and Exp-B with same SNR value were pieced together. Then the power spectral analysis was conducted on corresponding four final verification series and the original model ensemble mean series (Fig 2). For the cases with SNR of 1.0 and 0.5, the reconstruction series exhibit significant (90%) low-frequency signals, which cannot be recognized in the original model results (left panel in Fig 2) as well as the cases with SNR of 0.1 and 0.25. To sum up, the PPE really provides more insights into MDVM method proposed in this paper. These results could imply that the MDVM method possesses the ability to preserve more low-frequency variability, or at any rate won’t cause the reduction of low-frequency variability.

**Table 1:** Statistics of split validation results for the pseudo-proxy experiment. Exp-A: 851–1500 AD for calibration and 1501–2000 AD for validation; Exp-B: 1501–2000 AD for calibration and 851–1500 AD for validation; LF-Std-Ratio: the ratio of standard deviation of reconstructed centennial variability to simulated counterpart; Uncer-LF: the uncertainty of variability on centennial scale.

Experiments	SNR	RE	CE	RMSE	LF-Std-Ratio	Uncer-LF
Exp-A	0.1	0.957	0.815	0.140	0.885	0.0711
	0.25	0.975	0.882	0.107	0.872	0.0614
	0.5	0.986	0.939	0.081	0.936	0.0566
	1.0	0.983	0.927	0.086	0.947	0.0596
Exp-B	0.1	0.975	0.743	0.099	1.067	0.0696
	0.25	0.975	0.793	0.098	1.170	0.0567
	0.5	0.973	0.825	0.089	1.165	0.0532
	1.0	0.976	0.813	0.096	1.199	0.0505

**Fig 3:**
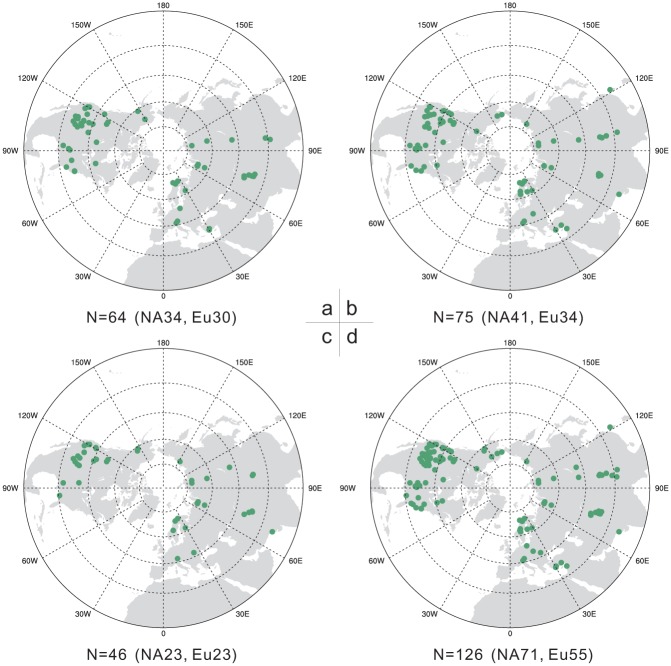
Spatial distribution of the screened tree-ring database used for the extratropical Northern Hemisphere temperature reconstruction during the last millennium on decadal (a), multi-decadal (b) and centennial (c) scale, respectively, as well as the distribution of total database (d) used on the three timescales. The total amount of tree-ring records was also given under each subgraph, and the number in brackets denoted the amount for the continental North America (NA) and Eurasia (Eu), respectively.

As shown in Table 2, the validation results of MDVM reconstruction showed positive values in RE and CE exceeding the threshold values at 95% significance level, and indicated that fairly reasonable skill was contained in the MDVM reconstruction [47, 48]. Eventually, 126 tree-ring records were screened out from 211 candidate records and utilized for the final reconstruction (detailed information was listed in S3 Table). Fig 3 showed the amount and spatial distribution of the tree-ring sites finally used for the extratropical NH temperature reconstruction on decadal, multi-decadal and centennial scale, respectively. It was found that, on each timescale, the location of selected tree-ring records was relatively well-distributed, and the quantity of proxy data derived from North America and Eurasia was also roughly balanced.

**Table 2:** Statistics of leave-one-out cross-validation results for the reconstructed composite series with CRU during the common period 1850–2000 AD. The threshold values at 95% significance level were estimated using 5000 member Monte Carlo simulations.

Metrics for validation	RE	CE	Uncertainty
95% significance level	0.522	0.317	0.211
Validation for MDVM	0.843	0.776	0.140

RE, the reduction of error; CE, coefficient of efficiency.

### Temperature reconstruction

**Fig 4:**
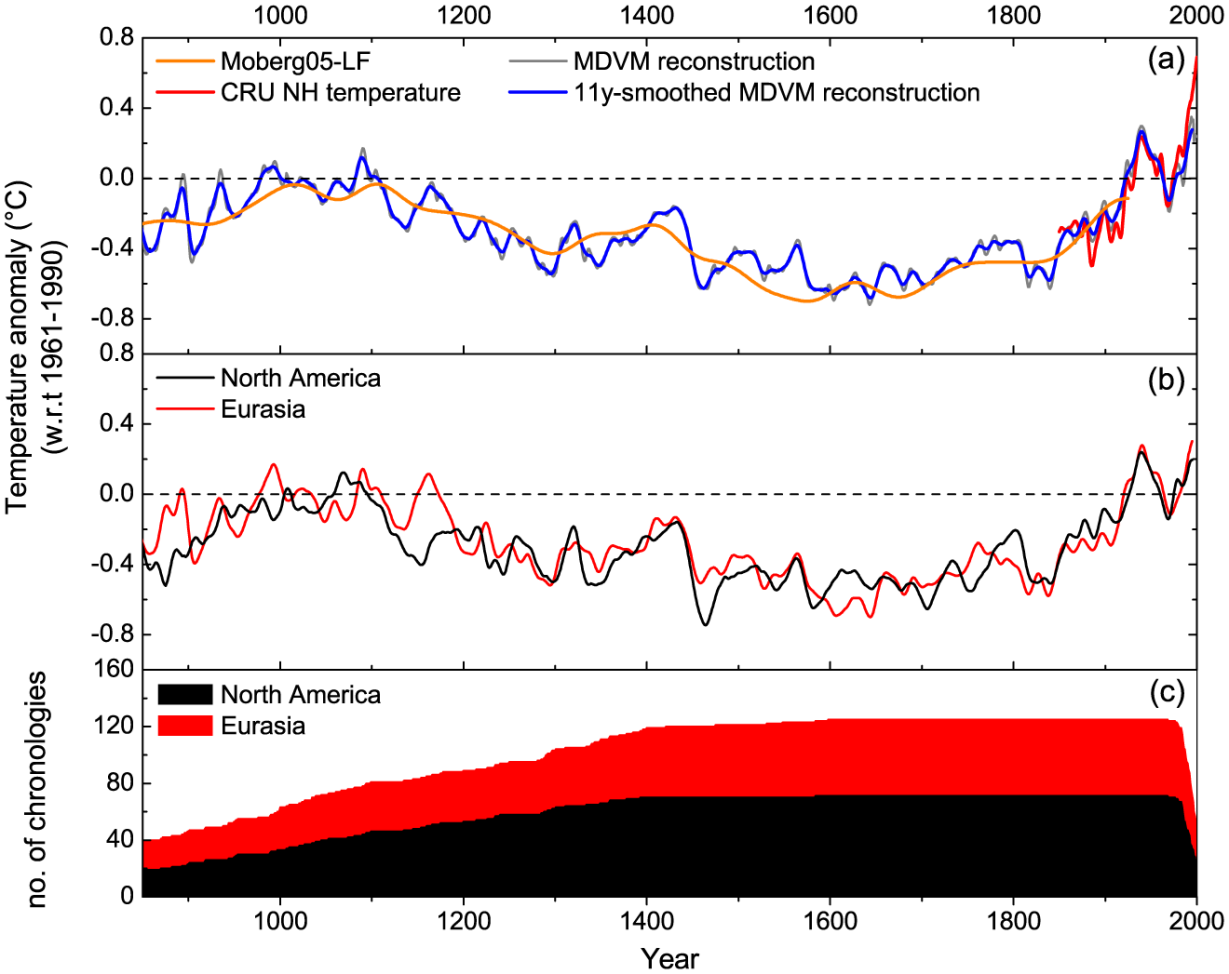
(a) Comparison between the benchmark series (CRU NH mean temperature and Moberg05-LF) and the reconstruction based on MDVM method. (b) Comparison of the reconstructions using different continental data for the extratropical Northern Hemisphere mean temperature. (c) The number of chronologies used for the reconstruction. All the smoothed series were 11-year running averaged. Temperatures were expressed as anomalies with respect to 1961–1990 AD.

Based on the MDVM method, the variation of the extratropical NH mean temperature during the past millennium was reconstructed and expressed as anomalies with respect to the period 1961–1990, as well as the comparison with benchmark series (Moberg05-LF and CRU) was shown (Fig 4a). The MDVM reconstruction agreed well with CRU series in decadal to multi-decadal scale. Nevertheless, at the end of the 20^th^ century, obvious discrepancy between MDVM reconstruction and CRU was found, which was probably due to the sharp decreasing of the chronologies’ total number in recent decade. The MDVM reconstruction captured evident low-frequency information and clearly indicated three typical epochs, i.e. 20^th^ century warming, LIA and MWP. The MDVM reconstruction exhibited an accelerated warming trend (0.038°C/decade) over recent 150 years. Pronounced LIA cumulating in 1450–1850 AD was depicted with the 17^th^ century being about 0.587°C below the average in 1961–1990 AD. Moderate warming episode of the MWP was observed over the interval 950–1150 AD with two warm peaks around AD 1000 and 1100. Based on the MDVM reconstruction in the past millennium, the coldest decades appeared in the 1640s, 1600s and 1580s, whereas the warmest decades occurred in the 1990s, 1940s and 1930s (Table 3).

**Table 3:** The top 3 coldest and warmest decades indicated by MDVM reconstruction for the extratropical Northern Hemisphere temperature anomaly with respect to the period 1961–1990 AD.

	Coldest			Warmest		
Decade	1640s	1600s	1580s	1990s	1940s	1930s
Anomaly	-0.685	-0.661	-0.634	0.281	0.214	0.188

Comparison of the reconstructions separately using different continental data for the extratropical NH mean temperature was also additionally exhibited (Fig 4b). It was found that the North America and Eurasia composites cohered well in respect of the general trend, but there were also some significant differences, such as those in the late twelfth century and early seventeenth century. Additionally, the North America composite showed much stronger cooling in the late fifteenth century, which was presumably related to volcanic eruptions. On the decadal scale, the North America composite displayed higher correlation (*r* = 0.732) with the CRU benchmark series than the Eurasia (*r* = 0.600). However, on the centennial scale, it exhibited weaker correlation (*r* = 0.754) with Moberg05-LF series than the Eurasia (*r* = 0.850). Therefore, tree-ring data from the North America probably made comparatively more contributions to the decadal variability of the extratropical NH mean temperature reconstruction, whereas data from the Eurasia was dedicated more to the centennial variability of the final reconstruction.

## Discussion

### Comparison with possible related external forcing

**Fig 5:**
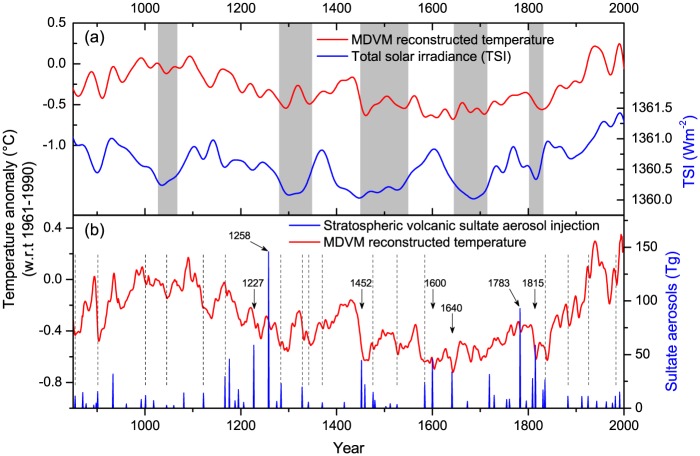
Comparison between MDVM reconstructed temperature (red lines) and the variations of external forcing. (a) Reconstructed total solar irradiance variations (blue line) obtained from the references [49, 50]. The smoothed lines were 30-year low-pass filtered and shadings denoted the timing of great solar minima. (b) Variations of reconstructed total stratospheric volcanic sulfate aerosol injection (blue bars) in Northern Hemisphere obtained from the references [57].

The comparison between MDVM reconstructed temperature and the variation of external forcing (solar activity and volcanic activity) is shown in Fig 5. The smoothed MDVM reconstruction exhibited a general agreement with the variation of the reconstructed total solar irradiance (TSI) [49, 50], and the correlation between the two series during the common period 849–2000 AD was significant (r = 0.498, edf = 34, p<0.01). Specially, the records shared high correlation coefficients in the epochs of the solar maximum (i.e. during the Medieval and Modern age), but poor correlation around 1500–1700 AD when the Spörer Minimum and Maunder Minimum occurred. It was similar to some other dendrochronological researches concerning the relation with solar activity [51–53]. The relatively cold conditions between the two warm peaks around AD 1000 and 1100 seemed to be related to the Oort Minimum [54]. However, there was an obvious discrepancy during the mid-16^th^ to mid-17^th^ century, i.e. relatively cold conditions corresponded to evidently high status of TSI, which was also observed in several other temperature reconstructions at regional (e.g. [23, 52, 55, 56]) or hemispheric scale (e.g. [4, 8, 12, 22]). It suggests that other external forcings exerted considerable influence on the low-frequency variability of past temperature besides the solar activity.

In Fig 5b, the MDVM temperature reconstruction was also compared with the volcano forcing time series in NH established in previous research [57]. It was found that the seven large volcanic eruptions, i.e. Tambora (1815), Laki (1783), Komaga-take (1640), Huaynaputina (1600), Kuwae (1452), Samalas (1258) and Reykjanes (1226/27), were associated with subsequently persistent shifts toward lower average temperatures in the MDVM reconstructions [58, 59]. Moreover, series of moderate and relatively-weak volcanic eruptions also coincided with significant drops of the reconstructed temperatures, such as in the years indicated by the dashed lines in Fig 5b (i.e. AD 1982, 1925, 1883, 1835, 1584, 1526, 1476, 1370, 1341, 1328, 1284, 1167, 1122, 1045, 1001, 901 and 854). These footprints of multiple volcanic eruptions in MDVM reconstruction probably resulted from relatively fine spatial coverage of the tree-ring network based on this novel method of separating timescales.

Therefore, the temperature reconstructions based on the MDVM method agreed well in general with the characteristic variations of the solar and volcanic forcings. It is quite plausible that the long-term climate variations in the past millennium have been largely linked to the periodical solar activity, and people usually look at the Maunder Minimum to explain the LIA [60–62]. However, some researchers argued that solar forcing probably had a minor effect on the climate change over the past 1000 years, and volcanic eruptions seemed to be an important driver for the climate particularly during the LIA [63, 64]. It was also reported that the abrupt onset of the LIA was likely triggered by a succession of strong volcanic eruptions and sustained by sea-ice/ocean feedbacks [65]. According to mainstream opinions, the LIA type events were probably attributed to a combination of solar minima and volcanic eruptions [66, 67].

### Comparison with reconstructions by different methods based on same dataset

**Fig 6:**
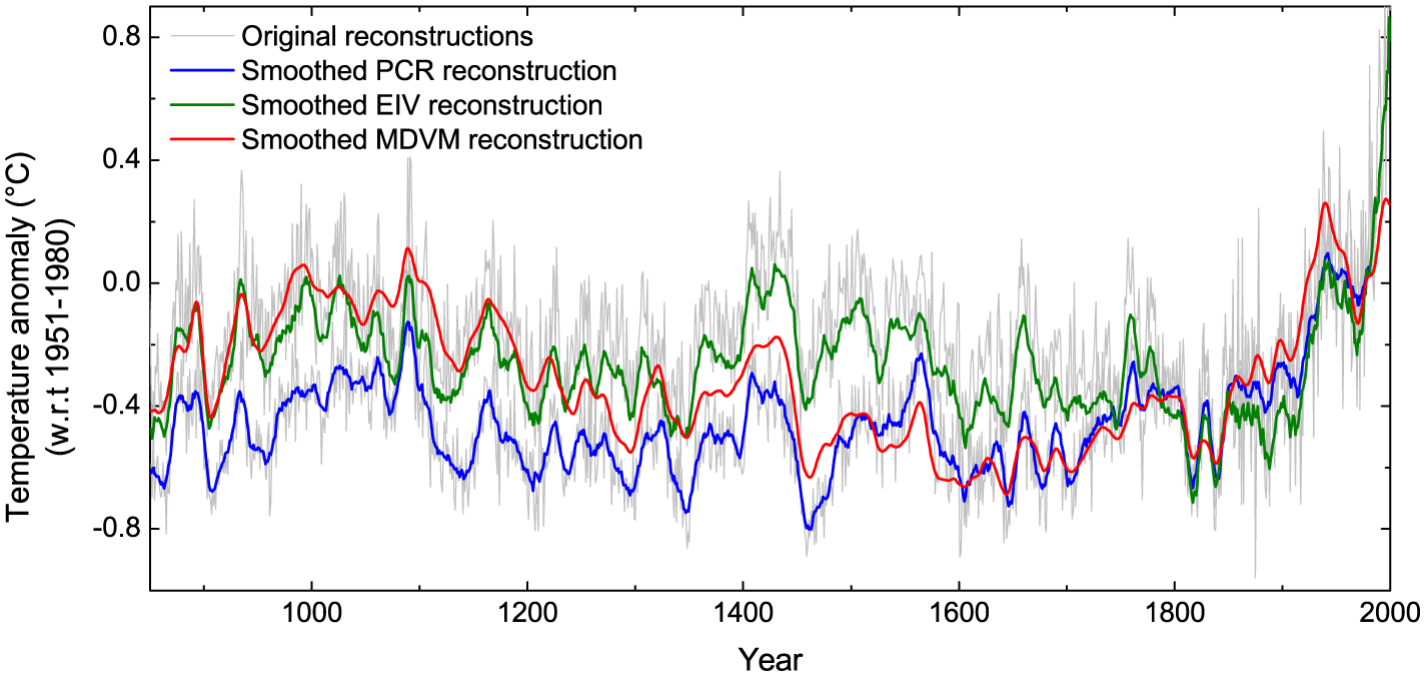
Comparison of extratropical Northern Hemisphere temperature reconstructions over the last millennium utilizing three statistical approaches, i.e. PCR, EIV, and MDVM, respectively. Temperatures were expressed as anomalies with respect to 1951–1980 AD. Bold lines were smoothed by 11-year moving average.

In order to highlight the performance of different methods used for large-scale temperature reconstructions, MDVM reconstruction was compared with other two versions of extratropical NH temperature reconstructions over the last millennium. They were based on the same tree-ring dataset but utilized three different statistical approaches, i.e. MDVM, principal components regression (PCR), and RegEM-based EIV (for more detailed information, please refer to reference [9]), respectively. In the screening procedure, employing the same criteria on threshold of the correlation analysis (i.e. being significant at a 95% level of confidence) which was used in MDVM reconstruction, finally only 37 and 69 tree-ring records with relatively sparse spatial distribution (not shown) were respectively utilized for the PCR and EIV reconstruction. The three versions of reconstructions were scaled by the individual mean during the period 1951–1980 AD (Fig 6). Although they all portrayed a persistent and unprecedented warming trend in the 20^th^ century, the low-frequency variability during the LIA and MWP were distinctly different. Obviously, the warming depicted in PCR reconstruction around the 11^th^ century was relatively marginal. The EIV reconstruction appeared to underestimate the cooling during the LIA particularly in the 17^th^ century despite comparable warming in the MWP relative to the MDVM reconstruction. Overall, the MDVM reconstruction exhibited more significant low-frequency variability in the past millennium, mainly resulting from the screening and calibration with the benchmark series of Moberg05-LF on centennial scale. Additionally, the spectral analysis results (not shown) indicated that about 80–90 year quasi-cycle was only significantly identified in the MDVM reconstruction at 99% confidence level This 80–90 years quasi-cycle is likely a natural cycle, which is probably forced by a centennial cycle of solar activity (i.e. Gleissberg cycle) [68, 69]. Also, some other studies indicated that reconstructions based on the PCR approach were prone to underestimate the amplitude of the low-frequency variability, whereas the EIV seemed to exaggerate the variability in some timespan [5, 70]. Based on the above comparisons, the MDVM method seemed to be more effective than PCR and EIV approach in large-scale reconstruction using pure tree-ring data, as it made use of more proxy records on different timescales and extracted more evident low-frequency variability.

### Comparison with previous reconstructions

**Fig 7:**
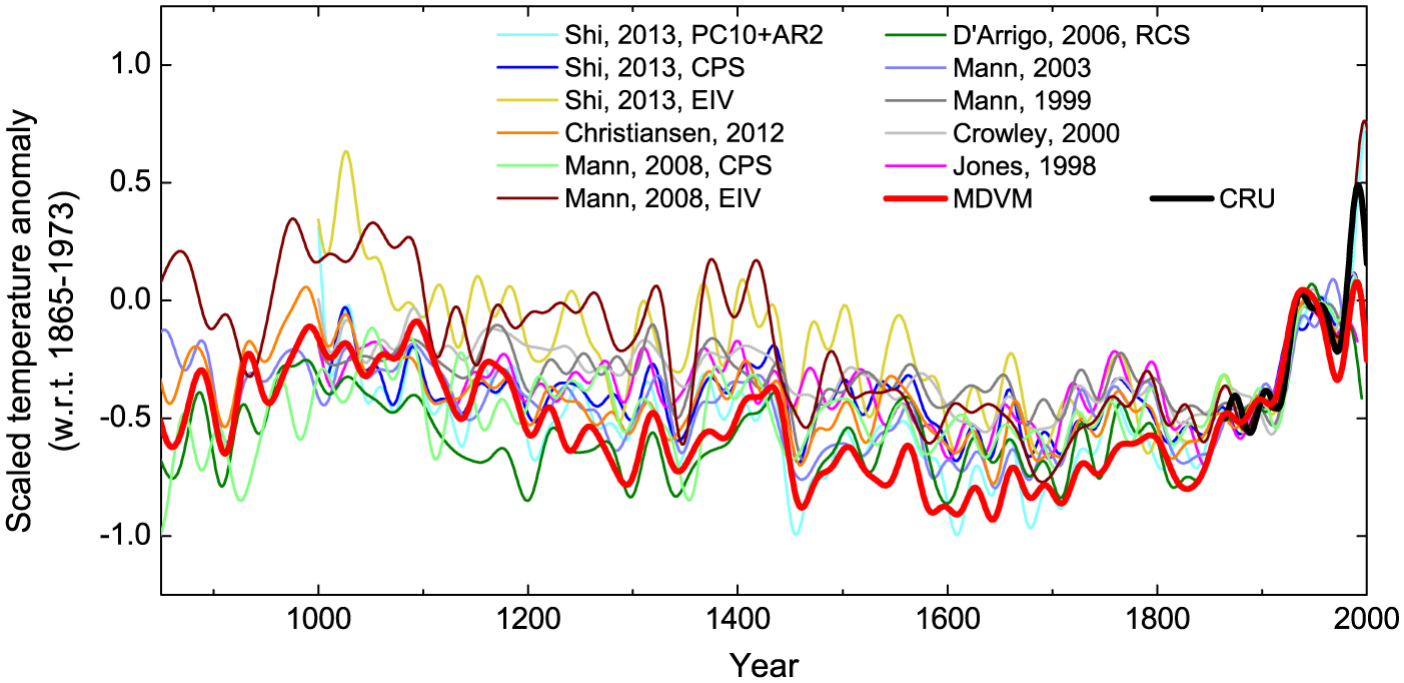
Comparison of MDVM reconstruction in this study with previous reconstructions for Northern Hemisphere mean temperature. All reconstructions were 30-year low-pass filtered, and scaled to the smoothed instrumental series by the variance and mean over the common period 1865–1973 AD.

**Fig 8:**
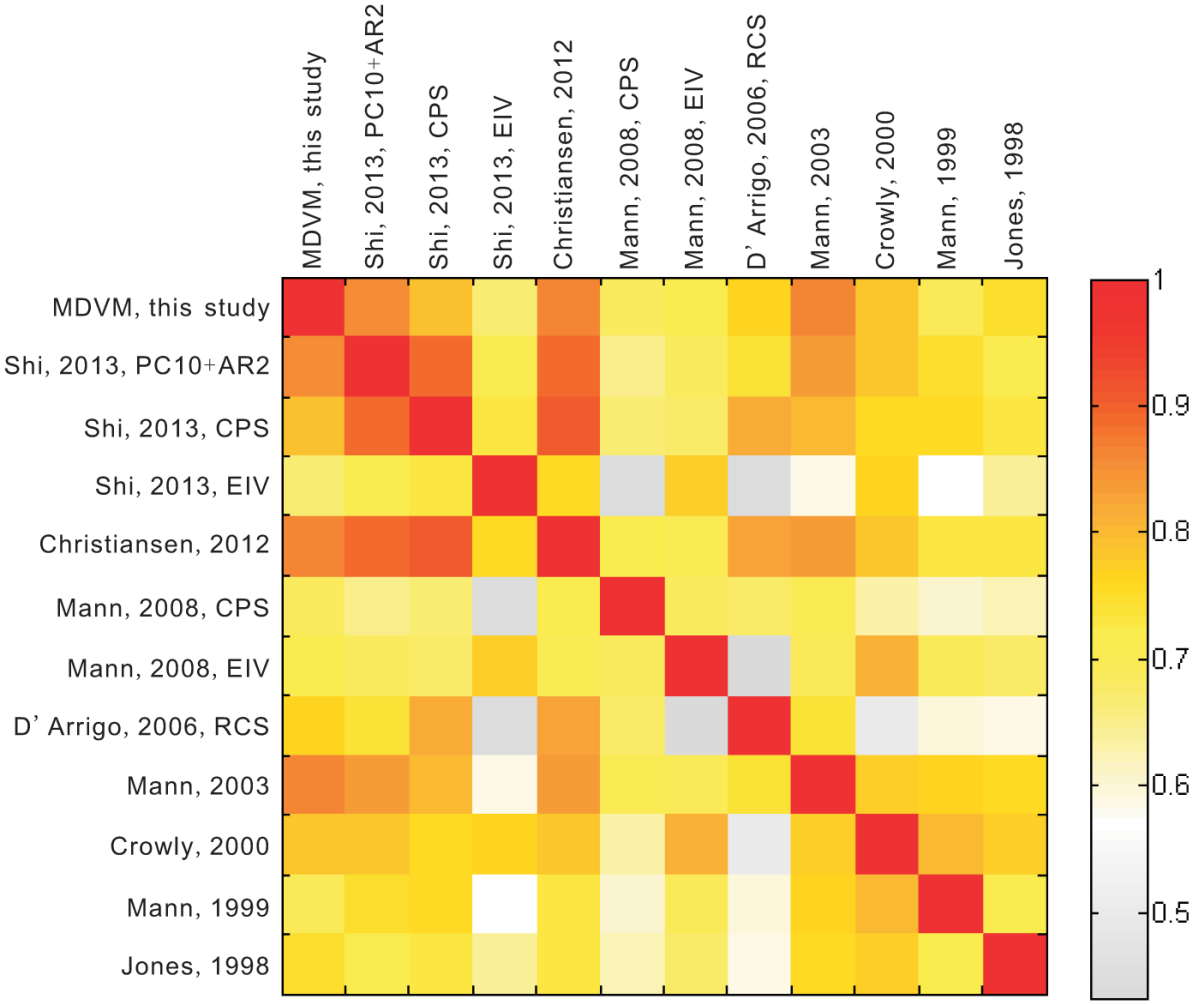
Color map of the correlation coefficient matrix for Northern Hemisphere temperature reconstructions (30-year low-pass filtered) over the common period 1000–1973 AD.

Our MDVM reconstruction was also compared to previous versions [1, 3–5, 7, 9, 21, 71] of reconstructed NH mean temperature (Fig 7). To facilitate the comparison, all reconstructions were 30-year low-pass filtered, and scaled to the smoothed instrumental series by the variance and mean over the common period 1865–1973 AD. It was found that our reconstruction was very similar to those from other studies in terms of the multi-decadal variability. The MDVM reconstruction captured much larger cooling during the LIA relative to the reference period, but also comparable warming signals in the MWP. In particular, the low-frequency variability indicated by MDVM reconstruction was more evident than the two reconstructions [3, 4] based solely on tree-ring data. It appeared that for the NH, the LIA maximum cooling occurred mainly centering in 17^th^ century, and the recent warming in 20^th^ century seemed to be unprecedented over the past millennium. Fig 8 presents the correlation coefficient matrix for all above NH temperature reconstructions (30-year low-pass filtered) over the common period 1000–1973. The correlation coefficients between the MDVM series and other reconstructions were relatively high as a whole, without any values lower than 0.6. These results further illustrated that MDVM reconstruction was in good agreement with others particularly in terms of the multi-decadal variability.

### Comparison with the results of climate model simulation

**Fig 9:**
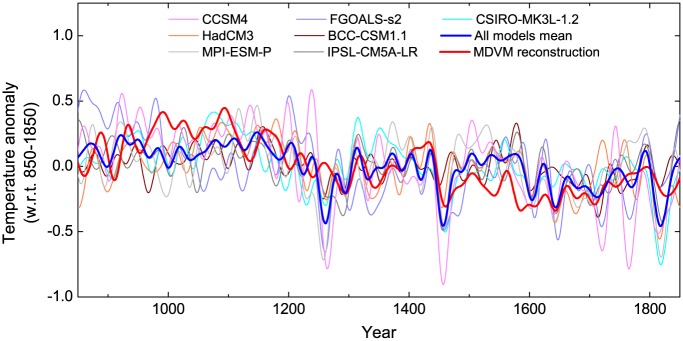
Comparison of MDVM reconstruction in this study with mode simulated Northern Hemisphere temperatures for the last millennium. All series were 30-year low-pass filtered, and scaled by the mean over the common period 850–1850 AD.

The MDVM reconstruction was also compared with outputs of climate model simulations over the period 850–1850 AD (Fig 9). It was found that our reconstruction generally agreed well with the model results on the multi-decadal scale, albeit with some differences. In addition, MDVM reconstruction showed much larger amplitude of low-frequency variability than model simulations. It appeared that most of the model simulations were prone to underestimate the warming during the MWP, which was also revealed by some other studies [4, 5]. Focusing on the strongest eruption of the past millennium (i.e. eruption in 1258 AD), it was obvious that the cooling effect of this eruption in MDVM reconstruction was much more muted than the analogous cooling indicated in the climate model simulations considered in this study. This discrepancy generally existed in other NH temperature reconstructions [72, 73] based at least partly on tree rings and it has aroused wide concern in recent years. Some researchers mainly attributed this disagreement to the misdating of the chronology which was caused by “stand-wide” missing rings near latitudinal tree line responding to the severely super eruptions [74]. However, several researchers have demonstrated that the underestimation of volcanic cooling in the tree-ring record was probably unrelated to any misdating [75–77]. This discrepancy seemed to arise from some other issues, such as the spatial distribution of tree-ring network, the seasonality of growth and climate response, and the lagged effects due to biological persistence, which all potentially affected the process of analyzing the climate signature of volcanic eruptions in the large-scale proxy-based reconstructions [75, 76].

## Conclusion

We introduced a novel method termed “MDVM”, which was a combination of the EEMD and variance matching techniques. This method allowed proxy data to take decomposition, screening, and calibration on separating timescales. Utilizing the MDVM method, a new reconstruction of the extratropical NH mean temperature for the period 850–2000 AD was established. 126 records with relatively fine spatial coverage were finally screened out and utilized to reconstruct temperature variability longer than decadal scale. The MDVM reconstruction depicted significant low-frequency variability in the past millennium with evident LIA and MWP. Temperature in 20^th^ century was likely unprecedented in an 1150-year context. Additionally, the MDVM reconstruction covaried broadly with changes in natural radiative forcing, and particularly showed distinct footprints of multiple volcanic eruptions in the last millennium. The comparisons with other versions of NH temperature reconstructions (using same dataset by different approaches, and previous versions developed by other people), as well as some model simulation data, further demonstrated the efficiency of MDVM method on studying large-scale and low-frequency climate signals using pure tree-ring data.

## Supporting Information

S1 Text Details of the Monte Carlo simulation. (PDF)

S1 Fig Comparison between the benchmark series (i.e. instrumental CRU variation and low-frequency variability obtained from reference [8]) and the reconstructed series based on tree-ring data by MDVM method on different timescales. The common period for decadal, multi-decadal and composite series spanned in 1850–2000 AD, whereas the period for centennial series spanned in 800–1925 AD.
(PDF)

S1 Table Mean of threshold values for each tree-ring component on decadal, multi-decadal and centennial scale, estimated by Monte Carlo simulation (5000 trials, Bootstrap resampling 400 times, at 95% level of confidence). (PDF)

S2 Table Statistics of scaling and calibrating on decadal, multi-decadal and centennial scale, respectively. (PDF)

S3 Table List of the 126 tree-ring records used for the MDVM extratropical Northern Hemisphere mean temperature reconstruction on separating timescales. (D, decadal scale; MD, multi-decadal scale; C, centennial scale). Most was ring-width data, and * denoted a few records of maximum latewood density.
(PDF)

## References

[1] Jones PD, Briffa KR, Barnett TP, Tett SFB. High-resolution palaeoclimatic records for the last millennium: interpretation, integration and comparison with General Circulation Model control-run temperatures. Holocene. 1998; 8: 455–471.

[2] Mann ME, Bradley RS, Hughes MK. Global-scale temperature patterns and climate forcing over the past six centuries. Nature. 1998; 392: 779–787.

[3] Mann ME, Bradley RS, Hughes MK. Northern hemisphere temperatures during the past millennium: inferences, uncertainties and limitations. Geophys Res Lett. 1999; 26: 759–762.

[4] D'Arrigo R, Wilson R, Jacoby G. On the long-term context for late twentieth century warming. J Geophys Res. 2006; 111: D03103.

[5] Shi F, Yang B, Mairesse A, Gunten Lv, Li J, Bräuning A, et al Northern Hemisphere temperature reconstruction during the last millennium using multiple annual proxies. Climate Research. 2013; 56: 231–244.

[6] Christiansen B, Ljungqvist FC. Reconstruction of the extratropical NH mean temperature over the last millennium with a method that preserves low-frequency variability. J Climate. 2011; 24: 6013–6034.

[7] Christiansen B, Ljungqvist FC. The extra-tropical Northern Hemisphere temperature in the last two millennia: reconstructions of low-frequency variability. Clim Past. 2012; 8: 765–786.

[8] Moberg A, Sonechkin DM, Holmgren K, Datsenko NM, Karlen W. Highly variable Northern Hemisphere temperatures reconstructed from low- and high-resolution proxy data. Nature. 2005; 433: 613–617. 15703742 10.1038/nature03265

[9] Mann ME, Zhang ZH, Hughes MK, Bradley RS, Miller SK, Rutherford S, et al Proxy-based reconstructions of hemispheric and global surface temperature variations over the past two millennia. Proc Natl Acad Sci USA. 2008; 105: 13252–13257. 10.1073/pnas.0805721105 18765811 PMC2527990

[10] Esper J, Cook ER, Schweingruber FH. Low-frequency signals in long tree-ring chronologies for reconstructing past temperature variability. Science. 2002; 295: 2250–2253. 11910106 10.1126/science.1066208

[11] Cook ER, Esper J, D'Arrigo RD. Extra-tropical Northern Hemisphere land temperature variability over the past 1000 years. Quaternary Sci Rev. 2004; 23: 2063–2074.

[12] Ljungqvist FC. A new reconstruction of temperature variability in the extra-tropical Northern Hemisphere during the last two millennia. Geogr Ann A. 2010; 92: 339–351.

[13] Jones PD, Mann ME. Climate over past millennia. Rev Geophys. 2004; 42: RG2002.

[14] Blaauw M, Christen JA, Mauquoy D, van der Plicht J, Bennett KD. Testing the timing of radiocarbon-dated events between proxy archives. Holocene. 2007; 17: 283–288.

[15] Esper J, Frank DC, Wilson RJS, Briffa KR. Effect of scaling and regression on reconstructed temperature amplitude for the past millennium. Geophys Res Lett. 2005; 32: L07711.

[16] Björklund JA, Gunnarson BE, Krusic PJ, Grudd H, Josefsson T, Östlund L, et al Advances towards improved low-frequency tree-ring reconstructions, using an updated Pinus sylvestris L. MXD network from the Scandinavian Mountains. Theor Appl Climatol. 2013; 113: 697–710.

[17] Briffa KR, Jones PD, Bartholin TS, Eckstein D, Schweingruber FH, Karlen W, et al Fennoscandian summers from AD 500: temperature changes on short and long timescales. Clim Dynam. 1992; 7: 111–119.

[18] Melvin TM, Briffa KR. A "signal-free" approach to dendroclimatic standardisation. Dendrochronologia. 2008; 26: 71–86.

[19] Cook ER, Briffa KR, Meko DM, Graybill DA, Funkhouser G. The 'segment length curse' in long tree-ring chronology development for palaeoclimatic studies. Holocene. 1995; 5: 229–237.

[20] Rutherford S, Mann ME, Osborn TJ, Bradley RS, Briffa KR, Hughes MK, et al Proxy-based Northern Hemisphere surface temperature reconstructions: Sensitivity to method, predictor network, target season, and target domain. J Climate. 2005; 18: 2308–2329.

[21] Mann ME, Jones PD. Global surface temperatures over the past two millennia. Geophys Res Lett. 2003; 30: L1820.

[22] Hegerl GC, Crowley TJ, Allen M, Hyde WT, Pollack HN, Smerdon J, et al Detection of human influence on a new, validated 1500-year temperature reconstruction. J Climate. 2007; 20: 650–666.

[23] Yang FM, Wang N, Shi F, Ljungqvist FC, Wang SG, Fan ZX, et al Multi-proxy temperature reconstruction from the West Qinling Mountains, China, for the past 500 years. Plos One. 2013; 8: e57638 10.1371/journal.pone.0057638 23451254 PMC3579785

[24] Christiansen B, Schmith T, Thejll P. A Surrogate Ensemble Study of Climate Reconstruction Methods: Stochasticity and Robustness. J Climate. 2009; 22: 951–976.

[25] Jones PD, Lister DH, Osborn TJ, Harpham C, Salmon M, Morice CP. Hemispheric and large-scale land-surface air temperature variations: An extensive revision and an update to 2010. J Geophys Res. 2012; 117: D05127.

[26] Taylor KE, Stouffer RJ, Meehl GA. An overview of CMIP5 and the experiment design. B Am Meteorol Soc. 2012; 93: 485–498.

[27] Gent PR, Danabasoglu G, Donner LJ, Holland MM, Hunke EC, Jayne SR, et al The Community Climate System Model version 4. J Climate. 2011; 24: 4973–4991.

[28] Collins M, Tett SFB, Cooper C. The internal climate variability of HadCM3, a version of the Hadley Centre coupled model without flux adjustments. Clim Dynam. 2001; 17: 61–81.

[29] Jungclaus JH, Lorenz SJ, Timmreck C, Reick CH, Brovkin V, Six K, et al Climate and carbon-cycle variability over the last millennium. Clim Past. 2010; 6: 723–737.

[30] Bao Q, Lin P, Zhou T, Liu Y, Yu Y, Wu G, et al The Flexible Global Ocean-Atmosphere-Land system model, Spectral Version 2: FGOALS-s2. Adv Atmos Sci. 2013; 30: 561–576.

[31] Wu T, Yu R, Zhang F, Wang Z, Dong M, Wang L, et al The Beijing Climate Center atmospheric general circulation model: description and its performance for the present-day climate. Clim Dynam. 2010; 34: 123–147.

[32] Hourdin F, Foujols M-A, Codron F, Guemas V, Dufresne J-L, Bony S, et al Impact of the LMDZ atmospheric grid configuration on the climate and sensitivity of the IPSL-CM5A coupled model. Clim Dynam. 2013; 40: 2167–2192.

[33] Phipps SJ. The CSIRO Mk3L climate system model v1.2. Technical Report No 4. 2010; Antarctic Climate & Ecosystems CRC, Hobart, Tasmania, Australia,121pp., ISBN 978-1-921197-04-8.

[34] Fang K, Gou X, Chen F, Liu C, Davi N, Li J, et al Tree-ring based reconstruction of drought variability (1615–2009) in the Kongtong Mountain area, northern China. Glob Planet Chang. 2012; 80–81: 190–197.

[35] Briffa KR, Melvin TM, Osborn TJ, Hantemirov RM, Kirdyanov AV, Mazepa VS, et al Reassessing the evidence for tree-growth and inferred temperature change during the Common Era in Yamalia, northwest Siberia. Quaternary Sci Rev. 2013; 72: 83–107.

[36] Yang B, Qin C, Wang J, He M, Melvin TM, Osborn TJ, et al A 3,500-year tree-ring record of annual precipitation on the northeastern Tibetan Plateau. Proc Natl Acad Sci USA. 2014; 111: 2903–2908. 10.1073/pnas.1319238111 24516152 PMC3939907

[37] Wigley TML, Briffa KR, Jones PD. On the average value of correlated time series, with applications in dendroclimatology and hydrometeorology. J Clim Appl Meteorol. 1984; 23: 201–213.

[38] Wu Z, Huang NE. Ensemble Empirical Mode Decomposition: a noise-assisted data analysis method. Adv Adapt Data Anal. 2009; 01: 1–41.

[39] Wu Z, Huang N, Wallace J, Smoliak B, Chen X. On the time-varying trend in global-mean surface temperature. Clim Dynam. 2011; 37: 759–773.

[40] Ji F, Wu Z, Huang J, Chassignet EP. Evolution of land surface air temperature trend. Nat Clim Change. 2014; 4: 462–466.

[41] Vecchio A, Carbone V. Amplitude-frequency fluctuations of the seasonal cycle, temperature anomalies, and long-range persistence of climate records. Phys Rev E. 2010; 82: D66101. 10.1103/PhysRevE.82.066101 21230699

[42] Shi F, Yang B, von Gunten L, Qin C, Wang Z. Ensemble empirical mode decomposition for tree-ring climate reconstructions. Theor Appl Climatol. 2012; 109: 233–243.

[43] Shi F, Li J, Wilson RJS. A tree-ring reconstruction of the South Asian summer monsoon index over the past millennium. Scientific Reports. 2014; 4: 6739 10.1038/srep06739 25338702 PMC4206867

[44] Metropolis N. The beginning of the Monte Carlo method. Los Alamos Science. 1987; Special Issue dedicated to Stanislaw Ulam: 125–130.

[45] Michaelsen J. Cross-validation in statistical climate forecast models. J Clim Appl Meteorol. 1987; 26: 1589–1600.

[46] Mann ME, Rutherford S, Wahl E, Ammann C. Robustness of proxy-based climate field reconstruction methods. J Geophys Res. 2007; 112: D12109.

[47] Fritts HC. Tree rings and climate. Academic Press, New York 1976.

[48] Cook ER, Briffa KR, Jones PD. Spatial regression methods in dendroclimatology: A review and comparison of two techniques. Int J Climatol. 1994; 14: 379–402.

[49] Vieira LEA, Solanki SK. Evolution of the solar magnetic flux on time scales of years to millenia. Astron Astrophys. 2010; 509: A100.

[50] Krivova NA, Balmaceda L, Solanki SK. Reconstruction of solar total irradiance since 1700 from the surface magnetic flux. Astron Astrophys. 2007; 467: 335–346.

[51] Rigozo NR, da Silva HE, Nordemann DJR, Echer E, de Souza Echer MP, Prestes A. The Medieval and Modern Maximum solar activity imprints in tree ring data from Chile and stable isotope records from Antarctica and Peru. J Atmos Sol-terr Phy. 2008; 70: 1012–1024.

[52] Corona C, Guiot J, Edouard JL, Chalié F, Büntgen U, Nola P, et al Millennium-long summer temperature variations in the European Alps as reconstructed from tree rings. Clim Past. 2010; 6: 379–400.

[53] Rigozo NR, Prestes A, Nordemann DJR, da Silva HE, Souza Echer MP, Echer E. Solar maximum epoch imprints in tree-ring width from Passo Fundo, Brazil (1741–2004). J Atmos Sol-terr Phy. 2008; 70: 1025–1033.

[54] Steinhilber F, Jürg B. Solar activity—the past 1200 years. PAGES news. 2011; 19: 5–6.

[55] Büntgen U, Raible CC, Frank D, Helama S, Cunningham L, Hofer D, et al Causes and consequences of past and projected Scandinavian summer temperatures, 500–2100 AD. Plos One. 2011; 6: e25133 10.1371/journal.pone.0025133 21966436 PMC3178611

[56] Anchukaitis KJ, D'Arrigo RD, Andreu-Hayles L, Frank D, Verstege A, Curtis A, et al Tree-Ring-Reconstructed Summer Temperatures from Northwestern North America during the Last Nine Centuries. J Climate. 2013; 26: 3001–3012.

[57] Gao C, Robock A, Ammann C. Volcanic forcing of climate over the past 1500 years: An improved ice core-based index for climate models. J Geophys Res. 2008; 113: D23111.

[58] Briffa KR, Jones PD, Schweingruber FH, Osborn TJ. Influence of volcanic eruptions on Northern Hemisphere summer temperature over the past 600 years. Nature. 1998; 393: 450–455.

[59] Lavigne F, Degeai J-P, Komorowski J-C, Guillet S, Robert V, Lahitte P, et al Source of the great A.D. 1257 mystery eruption unveiled, Samalas volcano, Rinjani Volcanic Complex, Indonesia. Proc Natl Acad Sci USA. 2013; 110: 16742–16747. 10.1073/pnas.1307520110 24082132 PMC3801080

[60] Eddy JA. The Maunder Minimum. Science. 1976; 192: 1189–1202. 17771739 10.1126/science.192.4245.1189

[61] Gray LJ, Beer J, Geller M, Haigh JD, Lockwood M, Matthes K, et al Solar influences on climate. Rev Geophys. 2010; 48: RG4001.

[62] Swingedouw D, Terray L, Cassou C, Voldoire A, Salas-Mélia D, Servonnat J. Natural forcing of climate during the last millennium: fingerprint of solar variability. Clim Dynam. 2011; 36: 1349–1364.

[63] Schrijver CJ, Livingston WC, Woods TN, Mewaldt RA. The minimal solar activity in 2008–2009 and its implications for long-term climate modeling. Geophys Res Lett. 2011; 38: L06701.

[64] Schurer AP, Tett SFB, Hegerl GC. Small influence of solar variability on climate over the past millennium. Nat Geosci. 2014; 7: 104–108.

[65] Miller GH, Geirsdottir A, Zhong YF, Larsen DJ, Otto-Bliesner BL, Holland MM, et al Abrupt onset of the Little Ice Age triggered by volcanism and sustained by sea-ice/ocean feedbacks. Geophys Res Lett. 2012; 39: L02708.

[66] Büntgen U, Esper J, Frank DC, Nicolussi K, Schmidhalter M. A 1052-year tree-ring proxy for Alpine summer temperatures. Clim Dynam. 2005; 25: 141–153.

[67] Guiot J, Corona C, ESCARSEL members. Growing season temperatures in Europe and climate forcings over the past 1400 years. Plos One. 2010; 5: e9972 10.1371/journal.pone.0009972 20376366 PMC2848609

[68] Feynman J, Fougere PF. Eighty-year periodicity in solar-terrestrial penomena confirmed. J Geophys Res. 1984; 89: 3023–3027.

[69] Peristykh AN, Damon PE. Persistence of the Gleissberg 88-year solar cycle over the last ~12,000 years: Evidence from cosmogenic isotopes. Journal of Geophysical Research: Space Physics. 2003; 108: 1003.

[70] Riedwyl N, Küttel M, Luterbacher J, Wanner H. Comparison of climate field reconstruction techniques: application to Europe. Clim Dynam. 2009; 32: 381–395.

[71] Crowley TJ. Causes of climate change over the past 1000 years. Science. 2000; 289: 270–277. 10894770 10.1126/science.289.5477.270

[72] Masson-Delmotte V, Schulz M, Abe-Ouchi A, Beer J, Ganopolski A, Rouco JFG, et al Information from paleoclimate archives In: Climate Change 2013: The Physical Science Basis Contribution of Working Group I to the Fifth Assessment Report of the Intergovernmental Panel on Climate Change [Stocker TF, Qin D, Plattner G-K, Tignor M, Allen SK, Boschung J, Nauels A, Xia Y, Bex V and Midgley PM (eds)] Cambridge University Press, Cambridge, United Kingdom and New York, NY, USA 2013.

[73] Mann ME, Rutherford S, Schurer A, Tett SFB, Fuentes JD. Discrepancies between the modeled and proxy-reconstructed response to volcanic forcing over the past millennium: Implications and possible mechanisms. J Geophys Res. 2013; 118: 7617–7627.

[74] Mann ME, Fuentes JD, Rutherford S. Underestimation of volcanic cooling in tree-ring-based reconstructions of hemispheric temperatures. Nat Geosci. 2012; 5: 202–205.

[75] Anchukaitis KJ, Breitenmoser P, Briffa KR, Buchwal A, Büntgen U, Cook ER, et al Tree rings and volcanic cooling. Nat Geosci. 2012; 5: 836–837.

[76] D'Arrigo R, Wilson R, Anchukaitis KJ. Volcanic cooling signal in tree ring temperature records for the past millennium. J Geophys Res. 2013; 118: 9000–9010.

[77] Esper J, Büntgen U, Luterbacher J, Krusic PJ. Testing the hypothesis of post-volcanic missing rings in temperature sensitive dendrochronological data. Dendrochronologia. 2013; 31: 216–222.

