# Supplementary material for: The Extratropical Northern Hemisphere Temperature Reconstruction during the Last Millennium Based on a Novel Method
Source: PLoS One. 2016 Jan 11;11(1):e0146776. doi: 10.1371/journal.pone.0146776 (PMC4709040; doi:10.1371/journal.pone.0146776)
Supplement: S1 Text — (PDF) [file pone.0146776.s001.pdf]

**S1 Text. Details of the Monte Carlo simulation**

In this study, the Monte Carlo simulation was performed for two parts work. One was to determine the significant threshold values of correlation coefficient at different timescales, and the other was to give significant level for the RE and CE of reconstructed series. For the latter part, the MC method followed the same procedure in the research conducted by Mann et al., the details of which were given in that paper [9] and would not be stated here. As for the former part, the surrogate series are produced by perturbing the original series with white noise. Considering the way that the EMD/EEMD process would affect the perturbation, the standard deviation of surrogate series is comparable to the original data. The Monte Carlo simulated series have the same auto-correlations as the original series, since white noise, in theory, should not change the auto-correlation of original time series. As stated in the section of MDVM method (Stage 2), for each original series (tree-ring chronologies and CRU series), 5000 simulated series are produced. For one particular series out of 5000 simulated ones, EMD algorithm is performed to obtain the corresponding IMFs, and then according to the diagnostic dominating cycles of each IMF, all these IMFs of that particular Monte Carlo simulated series are reduced into four components. The same procedure applied to the CRU series. For understanding this process easier, we can regard the EEMD/EMD plus mode reducing as one particular process that one time series could be decomposed into four components with pre-defined typical timescale (inter-annual, decadal, multi-decadal and centennial scale). Consequently, for each chronology, we have 5000x3 components (decadal, multi-decadal and centennial, as the

inter-annual component has been discarded) of chronology series and 5000x2 components of CRU (decadal, multi-decadal) and one low-frequency time series (Moberg05-LF). We perform bootstrap process to calculate the correlation coefficient another 5000 times, which are finally sorted and the corresponding quantiles (95%) are used as the threshold values (95% confidence level). The bootstrap is to provide a mean component of CRU and the chronology from a 400 member subset. And for the low-frequency (centennial timescale) component, the values of correlation coefficient are calculated directly with Moberg05-LF series.
