# Supplementary material for: The Extratropical Northern Hemisphere Temperature Reconstruction during the Last Millennium Based on a Novel Method
Source: PLoS One. 2016 Jan 11;11(1):e0146776. doi: 10.1371/journal.pone.0146776 (PMC4709040; doi:10.1371/journal.pone.0146776)
Supplement: S1 Fig — The common period for decadal, multi-decadal and composite series spanned in 1850–2000 AD, whereas the period for centennial series spanned in 800–1925 AD. (PDF) [file pone.0146776.s002.pdf]

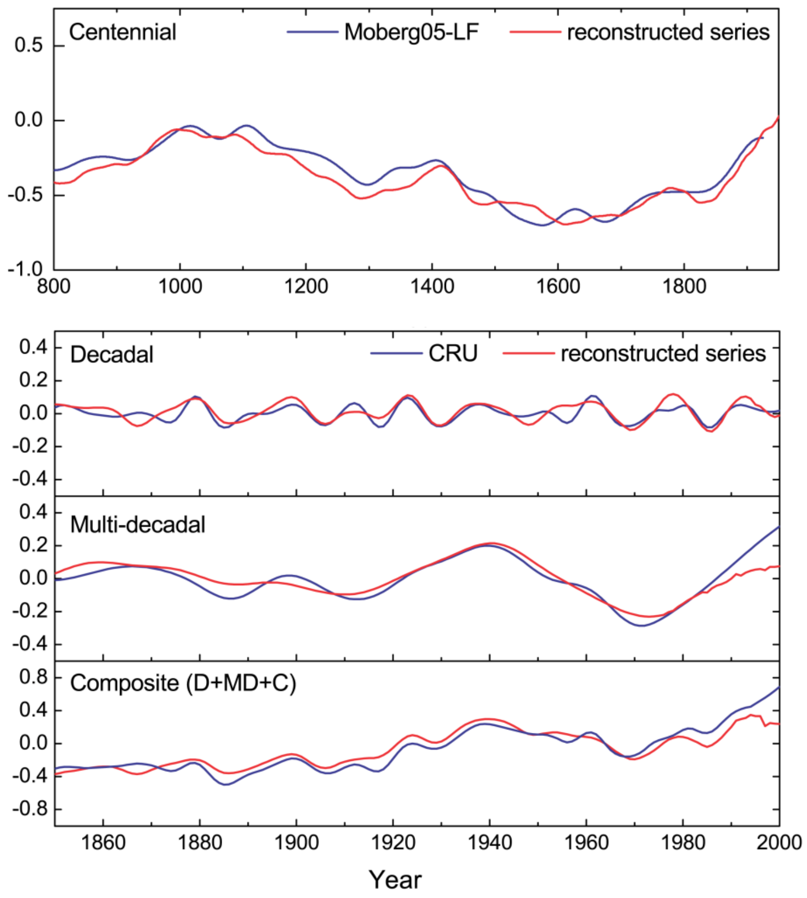

S1 Fig. Comparison between the benchmark series (i.e. instrumental CRU variation and low-frequency variability obtained from reference [8]) and the reconstructed series based on tree-ring data by MDVM method on different timescales. The common period for decadal, multi-decadal and composite series spanned in 1850-2000 AD, whereas the period for centennial series spanned in 800-1925 AD.
