# Supplementary material for: The Extratropical Northern Hemisphere Temperature Reconstruction during the Last Millennium Based on a Novel Method
Source: PLoS One. 2016 Jan 11;11(1):e0146776. doi: 10.1371/journal.pone.0146776 (PMC4709040; doi:10.1371/journal.pone.0146776)
Supplement: S1 Table — (PDF) [file pone.0146776.s003.pdf]

S1 Table. Mean of threshold values for each tree-ring component on decadal, multi-decadal and centennial scale, estimated by Monte Carlo simulation (5000 trials, Bootstrap resampling 400 times, at 95% level of confidence).

	Decadal	Multi-decadal	Centennial
Benchmark for correlation	CRU	CRU	low-frequency variation (reference [8])
Mean of the threshold values	0.258	0.476	0.403
