# Supplementary material for: The Extratropical Northern Hemisphere Temperature Reconstruction during the Last Millennium Based on a Novel Method
Source: PLoS One. 2016 Jan 11;11(1):e0146776. doi: 10.1371/journal.pone.0146776 (PMC4709040; doi:10.1371/journal.pone.0146776)
Supplement: S2 Table — (PDF) [file pone.0146776.s004.pdf]

S2 Table. Statistics of scaling and calibrating on decadal, multi-decadal and centennial scale, respectively.

	Decadal	Multi-decadal	Centennial	Composite
Period	1850-2000	1850-2000	133-1925	1850-2000
Scaler	0.351	0.914	0.605	--
r	0.771	0.888	0.862	0.875
