# Supplementary material for: The Extratropical Northern Hemisphere Temperature Reconstruction during the Last Millennium Based on a Novel Method
Source: PLoS One. 2016 Jan 11;11(1):e0146776. doi: 10.1371/journal.pone.0146776 (PMC4709040; doi:10.1371/journal.pone.0146776)
Supplement: S3 Table — Most was ring-width data, and * denoted a few records of maximum latewood density. (PDF) [file pone.0146776.s005.pdf]

S3 Table. List of the 126 tree-ring records used for the MDVM extratropical Northern Hemisphere mean temperature reconstruction on separating timescales (D, decadal scale; MD, multi-decadal scale; C, centennial scale). Most was ring-width data, and * denoted a few records of maximum latewood density.

#	Site	Lon	Lat	Begin year	End year	Reference	Scale
1	Noatak	67.07	-159.62	1094	1992	ITRDB, ak046	MD
2	Coastal Alaska	61.05	-146.98	691	2000	ITRDB, ak096	D, C
3	Sukak Pak	67.35	-149.47	1311	1979	ITRDB, ak106	D
4	Bayou Deview	34.95	-91.22	1145	1985	ITRDB, ar049	D, MD
5	Mayberry Slough	35.55	-91.25	1288	1990	ITRDB, ar052	MD
6	St. Francis Sunklands	35.63	-90.45	1360	1990	ITRDB, ar054	D, MD
7	Hemmed in Hollow	36.08	-93.30	1359	1992	ITRDB, ar055	MD
8	Flower Lake	36.77	-118.37	1175	1987	ITRDB, ca528	MD
9	Timber gap upper	36.45	-111.67	1099	1987	ITRDB, ca529	D, MD
10	Campito Mountain	37.50	-118.22	882	1983	ITRDB, ca533	C
11	Carson Pass East, Caples	38.70	-120.00	901	1999	ITRDB, ca630	D, C
12	Jackson Meadow Reservoir	39.52	-120.55	1035	1999	ITRDB, ca631	D, C
13	Boreal plateau	36.45	-118.55	1045	1992	ITRDB, ca636	MD
14	Crabtree	36.97	-118.62	954	1988	ITRDB, ca638	MD
15	Lac Duparquet	48.47	-79.28	1234	1987	ITRDB, cana106	D
16	Dividing Lake	45.4	-78.6	1068	1993	ITRDB, cana148	MD
17	Westward Lake	45.5	-78.78	1285	1993	ITRDB, cana149	MD
18	Assiniboine River Alluvial	49.33	-97.17	1349	1968	ITRDB, cana193	D, MD
19	Whirlpool Point	52.00	-116.45	831	1996	ITRDB, cana220	MD
20	Grays Creek pass	49.58	-116.68	822	1991	ITRDB, cana308	MD
21	Dulan	36.00	98.00	896	1993	ITRDB, chin006	MD
22	Delingha	37.43	98.05	451	2000	ITRDB, chin053	C
23	Almagre Mountain	38.77	-104.98	893	1983	ITRDB, co524	C
24	Hermit Lake	38.10	-105.63	1166	1983	ITRDB, co525	MD
25	Great Sand Dunes High	37.72	-105.47	1039	1995	ITRDB, co556	D, C
26	Lily Lake	40.32	-105.55	1074	1998	ITRDB, co572	MD
27	Land's End	39.00	-108.15	1301	2000	ITRDB, co580	D, MD
28	Wild Rose	39.02	-108.23	1154	2000	ITRDB, co631	D, MD
29	Trail Gulch update	39.72	-106.98	1160	2000	ITRDB, co649	D

30	Choctawhatchee River	30.45	-85.92	899	1992	ITRDB, fl001	C
31	Ebenezer Creek	32.35	-81.22	990	1985	ITRDB, ga003	MD
32	Kyrgyzstan High Hoc	40.17	72.58	1396	1995	ITRDB, kyrg005	D
33	Kyrgyzstan High Mur	40.17	72.58	1339	1995	ITRDB, kyrg007	D
34	Kursala Ridge	39.92	71.47	1281	1987	ITRDB, kyrg009	D
35	Sheveli Canyon	39.83	71.50	938	1987	ITRDB, kyrg011	D
36	Big Cypress State Park	32.25	-92.97	997	1988	ITRDB, la001	D, MD,
37	Besheri	34.23	36.03	1531	2000	ITRDB, leba004	MD
38	Leatherwood Creek	37.10	-91.53	1214	1990	ITRDB, mo043	MD
39	Grand River Basin	40.08	-93.58	1007	2000	ITRDB, mo047	MD, C
40	Solongotyn Davaa	48.30	98.93	819	1999	ITRDB, mong003	D, MD
41	Hentii Mountains	48.35	107.47	1024	2000	ITRDB, mong021	C
42	North Fork Ridge	45.30	-111.33	681	2000	ITRDB, mt111	C
43	Yellow Mountain Ridge 1	45.30	-111.32	806	1998	ITRDB, mt112	MD
44	Flint Creek range	46.28	-113.15	1010	1998	ITRDB, mt116	MD, C
45	Pintlers	46.02	-113.37	1584	2000	ITRDB, mt119	MD
46	Black River	34.32	-78.22	430	1985	ITRDB, nc008	MD
47	El Malpais National	34.97	-108.10	1	1992	ITRDB, nm572	D, C
48	El Malpais update	34.97	-108.18	502	2000	ITRDB, nm580	D
49	Mesa Alta	36.28	-106.62	1021	2000	ITRDB, nm584	D, C
50	Navajo Monument	36.70	-110.50	581	2000	ITRDB, nm588	D, C
51	Navajo Monument	36.70	-110.50	1	2000	ITRDB, nm589	D
52	Charleston Peak	36.27	-115.70	989	1984	ITRDB, nv510	MD
53	Pearl Peak	40.23	-115.53	392	1985	ITRDB, nv512	D, MD
54	Washington mount	38.90	-114.32	919	1983	ITRDB, nv513	MD, C
55	Hill 10842	38.93	-114.23	1	1984	ITRDB, nv516	MD, C
56	Lucky Horseshoe	37.87	-118.33	1081	2000	ITRDB, nv519	D
57	Washington mount	38.92	-114.32	1	2000	ITRDB, nv520	C
58	Frederick Butte	43.58	-120.45	870	1996	ITRDB, or060	MD, C
59	Horse Ridge	43.97	-121.07	830	1996	ITRDB, or061	MD
60	Table Rock, Arrow Gap	43.18	-120.90	530	1996	ITRDB, or062	C
61	Pakistan high Cha2	36.33	74.03	1250	1993	ITRDB, paki006	D, MD
62	Boibar Valley 2	36.58	75.42	1067	1990	ITRDB, paki010	MD, C
63	Boibar Valley 4	36.58	75.08	1343	1990	ITRDB, paki012	MD
64	Pakistan high Sat2	35.17	75.50	856	1993	ITRDB, paki015	D
65	Dashkin	35.45	74.78	1598	2000	ITRDB, paki025	MD
66	Mushfar Gilgit	35.50	74.08	1296	2000	ITRDB, paki031	D
67	East Pomerania	53.50	16.00	1035	1984	ITRDB, pola006	D
68	Four Holes Swamp	33.18	-80.42	1053	1984	ITRDB, sc004	D

69	Reno Gulch	43.90	-103.60	1393	1991	ITRDB, sd017	D, MD
70	Norris Basin Hawley	36.20	-84.00	1378	2000	ITRDB, tn031	D
71	Antalya	36.67	29.92	1370	1988	ITRDB, turk005	D, MD
72	Elmali	36.60	30.02	1093	2000	ITRDB, turk016	D
73	Goller	37.08	30.52	1421	2000	ITRDB, turk018	D
74	Neseli	37.20	34.47	1290	2000	ITRDB, turk042	MD
75	Cilan	24.53	121.38	1178	2000	ITRDB, tw001	MD
76	Mammoth Creek	37.65	-112.67	436	1985	ITRDB, ut509	MD
77	Nutter's Ridge	39.82	-110.67	1226	2000	ITRDB, ut526	D
78	Well's Draw	39.83	-110.17	952	2000	ITRDB, ut527	D
79	Beef Basin	37.93	-109.80	581	2000	ITRDB, ut529	C
80	Blackwater River	36.78	-76.88	944	1985	ITRDB, va021	D
81	Nottoway River	36.78	-77.13	1235	1984	ITRDB, va025	D, MD
82	Segelson Pass	48.35	-121.75	1334	1976	ITRDB, wa041	D, MD
83	Frying Pan Creek	46.88	-121.62	1292	1979	ITRDB, wa048	MD
84	Cedar/Moser/Bible Knobs	38.65	-79.37	1027	1998	ITRDB, wv005	MD
85	Mt. Everts includes	44.98	-110.67	1240	1999	ITRDB, wy027	D
86	McDougal Pass	42.80	-110.60	1090	1997	ITRDB, wy038	D
87	Delingha, China	33.75	98.75	976	2000	Zhang & Qiu (2007)	MD
88	Karakorum	36.37	74.99	618	1993	Esper et al. (2002)	C
89	Tien Shan	40.00	72.00	1000	1995	Esper et al. (2003)	C
90	Tien Shan & Karakorum	37.93	74.11	686	1999	Esper et al. (2007)	D, C
91	Northern Scandinavian	24.40	68.15	1	2000	Esper et al. (2012)	MD, C
92	Jämtland, Sweden	63.17	13.00	1	2000	Linderholm & Bräuning (2005)	C
93	Qamdo, China	31.12	97.03	1000	1994	Linderholm & Bräuning (2006)	D
94	Eastern Carpathians	47.20	25.25	994	2000	Popa & Kern (2009)	C
95	Lapland, Finland	69.00	25.00	1	2000	Helama et al. (2010)	D, MD,
96	Varparanta, Savonlinna	61.95	28.89	1	2000	Helama et al. (2012)	D, MD,
97	Lofoten-Vesteralen	69.14	16.52	1548	1989	Kirchhefer (2001)	D, MD
98	Idaho	44.42	-114.25	1135	1992	Biondi et al. (1999)	MD
99	Indigirka	70.53	148.15	1	1993	Moberg et al. (2006)	MD, C
100	Southern Colorado Plateau	35.20	-111.40	1	2000	Salzer & Kipfmueller (2005)	D
101	Yamalia, NW Siberia	66.85	68.4	1	2000	Briffa et al. (2013)	D, C
102	Polar Urals,	66.82	65.58	872	2000	Briffa et al. (2013)	D, MD
103	Polar Urals,	66.82	65.58	778	2000	Briffa et al. (2013) *	D, C
104	Northern Siberia	72.00	100.00	1400	1991	Briffa et al. (2001) *	D, MD
105	Coastal Alaska	62.00	-145.00	713	2000	D’Arrigo et al. (2006)	C
106	Tornetraesk	68.00	20.00	747	1980	D’Arrigo et al. (2006)	D, MD
107	Taymir	72.00	100.00	755	1997	D’Arrigo et al. (2006)	MD

108	Icefields	52.00	-115.00	918	1994	D'Arrigo et al. (2006)	D, MD
109	Polar Urals	67.00	66.00	944	1996	D'Arrigo et al. (2006)	D, MD
110	Central NWT	66.00	-120.00	1288	2000	D'Arrigo et al. (2006)	MD
111	NW North Alaska	67.00	-167.00	1297	2000	D'Arrigo et al. (2006)	MD
112	Jaemtland	64.00	14.00	1340	1978	D'Arrigo et al. (2006)	MD
113	Alps	46.00	10.00	1350	1995	D'Arrigo et al. (2006)	D, MD
114	Torneträsk	68.26	19.63	501	2000	Grudd (2008)	D, MD
115	Torneträsk	68.26	19.63	501	2000	Grudd (2008) *	D
116	Austrian Alps	46.88	11.32	1	2000	Büntgen et al. (2011)	D, MD,
117	Tatra, Slovakia	49.50	20.00	1061	2000	Büntgen et al. (2013)	MD
118	Jämtland, Central Sweden	62.27	13.38	1290	2000	Gunnarson et al. (2011) *	MD
119	Rockies, Canada	52.15	-117.15	950	1994	Luckman and Wilson (2005) *	D, C
120	Fennoscandia	62.50	22.50	1	1997	Briffa et al. (2008)	MD
121	Yamal, Russia	62.50	67.50	1	1996	Briffa et al. (2008)	D, MD,
122	Avam-Taimyr	62.50	102.50	1	2000	Briffa et al. (2008)	D, MD,
123	Eastern Taimyr	71.75	105.00	1	1996	Naurzbaev et al. (2002)	MD, C
124	Geza, Yunnan, China	28.37	99.77	1475	2000	Li et al. (2011)	MD
125	Bomi-Linzhi, Tibet	29.00	96.00	1385	2000	Zhu et al. (2011)	D
126	Wulan, Qinghai, China	37.03	98.65	1000	2000	Zhu et al. (2008)	MD, C

References referred in Table S3:

1. Zhang QB, Qiu HY. A millennium-long tree-ring chronology of *Sabina przewalskii* on northeastern Qinghai-Tibetan Plateau. Dendrochronologia. 2007; 24: 91-95.
2. Esper J, Schweingruber FH, Winiger M. 1300 years of climatic history for Western Central Asia inferred from tree-rings. Holocene. 2002; 12: 267-277.
3. Esper J, Shiyatov SG, Mazepa VS, Wilson RJS, Graybill DA, Funkhouser G. Temperature-sensitive Tien Shan tree ring chronologies show multi-centennial growth trends. Clim Dynam. 2003; 21: 699-706.
4. Esper J, Frank DC, Wilson RJS, Buntgen U, Treydte K. Uniform growth trends among central Asian low- and high-elevation juniper tree sites. Trees-Struct Funct. 2007; 21: 141-150.
5. Esper J, Büntgen U, Timonen M, Frank DC. Variability and extremes of northern Scandinavian summer temperatures over the past two millennia. Glob Planet Chang. 2012; 88-89: 1-9.
6. Linderholm HW, Gunnarson BE. Summer temperatuer variability in Central Scandinavia during the last 3600 years. Geogr Ann A. 2005; 87: 231-241.
7. Linderholm HW, Brauning A. Comparison of high-resolution climate proxies from the Tibetan Plateau and Scandinavia during the last millennium. Quat Int 2006; 154: 141-148.
8. Popa I, Kern Z. Long-term summer temperature reconstruction inferred from tree-ring records from the Eastern Carpathians. Clim Dynam. 2009; 32: 1107-1117.
9. Helama S, Fauria MM, Mielikäinen K, Timonen M, Eronen M. Sub-Milankovitch solar forcing of past climates: Mid and late Holocene perspectives. Geol Soc Am Bull 2010: 1981-1988.
10. Helama S, Bégin Y, Vartiainen M, Peltola H, Kolström T, Meriläinen J. Quantifications of

dendrochronological information from contrasting microdensitometric measuring circumstances of experimental wood samples. Appl Radiat Isot 2012; 70: 1014-1023.

11. Kirchhefer AJ. Reconstruction of summer temperatures from tree-rings of Scots pine (*Pinus sylvestris* L.) in coastal northern Norway. Holocene. 2001; 11: 41-52.
12. Biondi F, Perkins DL, Cayan DR, Hughes MK. July temperature during the second millennium reconstructed from Idaho tree rings. Geophys Res Lett. 1999; 26: 1445-1448.
13. Moberg A, Sonechkin DM, Holmgren K, Datsenko NM, Karlen W, Lauritzen S-E. Corrigendum: Highly variable Northern Hemisphere temperatures reconstructed from low- and high-resolution proxy data. Nature. 2006; 439: 1014.
14. Salzer MW, Kipfmueller KF. Reconstructed temperature and precipitation on a millennial timescale from tree-rings in the Southern Colorado Plateau, USA. Clim Change 2005; 70: 465-487.
15. Briffa KR, Melvin TM, Osborn TJ, Hantemirov RM, Kirdyanov AV, Mazepa VS, et al. Reassessing the evidence for tree-growth and inferred temperature change during the Common Era in Yamalia, northwest Siberia. Quaternary Sci Rev. 2013; 72: 83-107.
16. Briffa KR, Osborn TJ, Schweingruber FH, Harris IC, Jones PD, Shiyatov SG, et al. Low-frequency temperature variations from a northern tree ring density network. J Geophys Res 2001; 106: 2929-2941.
17. D'Arrigo R, Wilson R, Jacoby G. On the long-term context for late twentieth century warming. J Geophys Res. 2006; 111: D03103.
18. Grudd H. Torneträsk tree-ring width and density AD 500–2004: a test of climatic sensitivity and a new 1500-year reconstruction of north Fennoscandian summers. Clim Dynam. 2008; 31: 843-857.
19. Büntgen U, Tegel W, Nicolussi K, McCormick M, Frank D, Trouet V, et al. 2500 years of European climate variability and human susceptibility. Science. 2011: 578-582.
20. Büntgen U, Kyncl T, Ginzler C, Jacks DS, Esper J, Tegel W, et al. Filling the Eastern European gap in millennium-long temperature reconstructions. Proc Natl Acad Sci USA. 2013; 110: 1773-1778.
21. Gunnarson BE, Linderholm HW, Moberg A. Improving a tree-ring reconstruction from west-central Scandinavia: 900 years of warm-season temperatures. Clim Dynam. 2011; 36: 97-108.
22. Luckman BH, Wilson RJS. Summer temperatures in the Canadian Rockies during the last millennium: a revised record. Clim Dynam. 2005; 24: 131-144.
23. Briffa KR, Shishov VV, Melvin TM, Vaganov EA, Grudd H, Hantemirov RM, et al. Trends in recent temperature and radial tree growth spanning 2000 years across northwest Eurasia. Philos Trans R Soc Lond B Biol Sci. 2008; 363: 2269-2282.
24. Naurzbaev MM, Vaganov EA, Sidorova OV, Schweingruber FH. Summer temperatures in eastern Taimyr inferred from a 2427-year late-Holocene tree-ring chronology and earlier floating series. Holocene. 2002; 12: 727-736.
25. Li Z-S, Zhang Q-B, Ma K. Tree-ring reconstruction of summer temperature for A.D. 1475-2003 in the central Hengduan Mountains, Northwestern Yunnan, China. Clim Change 2011; 110: 455-467.
26. Zhu H-F, Shao X-M, Yin Z-Y, Xu P, Xu Y, Tian H. August temperature variability in the southeastern Tibetan Plateau since AD 1385 inferred from tree rings. Palaeogeogr Palaeoclimatol Palaeoecol 2011; 305: 84-92.
27. Zhu H, Zheng Y, Shao X, Liu X, Xu Y, Liang E. Millennial temperature reconstruction based on tree-ring widths of Qilian juniper from Wulan, Qinghai Province, China. Chin Sci Bull 2008; 53: 3914-3920.
